# Drug Reaction With Eosinophilia and Systemic Symptoms (DRESS) Syndrome With Predominant Renal Involvement and Cholestatic Liver Injury: A Case Report

**DOI:** 10.7759/cureus.108236

**Published:** 2026-05-04

**Authors:** Tamar Megrelishvili, Elene Saribekovi, Elene Pachkoria, Tamar Didbaridze, Ia Mikadze, Magda Rurua, Levan Ratiani

**Affiliations:** 1 Department of Infectious Diseases, The First University Clinic of Tbilisi State Medical University, Tbilisi, GEO; 2 Department of Infectious Diseases, Tbilisi State Medical University, Tbilisi, GEO; 3 Department of Microbiology, The First University Clinic of Tbilisi State Medical University, Tbilisi, GEO; 4 Department of Anesthesiology and Critical Care Medicine, The First University Clinic of Tbilisi State Medical University, Tbilisi, GEO

**Keywords:** acute renal injury, amoxicillin–clavulanate, cholestatic liver injury, corticosteroids, drug reaction with eosinophilia and systemic symptoms (dress) syndrome

## Abstract

Drug reaction with eosinophilia and systemic symptoms (DRESS) syndrome is a rare but potentially life-threatening drug-induced hypersensitivity reaction characterized by delayed onset and variable multi-organ involvement, which can complicate timely diagnosis. We report the case of a 75-year-old female who developed fever, generalized pruritic maculopapular rash, facial edema, lymphadenopathy, and acute kidney injury 10 days after completing a one-week course of amoxicillin-clavulanate for a respiratory infection. Laboratory evaluation revealed leukocytosis with neutrophilia, mild eosinophilia not meeting diagnostic threshold criteria, acute kidney injury with proteinuria and hematuria, and isolated cholestatic liver injury without significant ALT or AST elevation; atypical lymphocytosis was absent. Autoimmune serology and viral hepatitis screening were negative. Based on the RegiSCAR scoring system, the patient achieved a score of 7, consistent with a definite diagnosis of DRESS. Systemic corticosteroid therapy, alongside adjunctive topical treatment and antihistamines, led to progressive clinical and laboratory improvement. This case represents an atypical presentation of DRESS with predominantly renal involvement and isolated cholestatic liver injury, where the absence of typical hematologic manifestations posed a diagnostic challenge. Although viral reactivation, particularly HHV-6, is often implicated in DRESS pathogenesis, its role here remains unconfirmed due to the lack of virological testing. The case highlights the importance of considering DRESS in patients with delayed-onset cutaneous eruptions and unexplained acute kidney injury after drug exposure, even in the absence of classic hematologic features, as early recognition and appropriate management are crucial for favorable outcomes.

## Introduction

Drug reaction with eosinophilia and systemic symptoms (DRESS) syndrome is a potentially life-threatening, multisystem drug hypersensitivity reaction that typically develops days to weeks after the initiation or discontinuation of the offending medication [1]. Common causative agents include anticonvulsants, sulfonamides, allopurinol, vancomycin, and beta-lactam antibiotics. However, the culprit drug remains unidentified in approximately 10-20% of cases [2,3].

The estimated incidence of DRESS ranges from 1 in 10,000 to 1 in 100,000 individuals. However, many cases likely remain unrecognized because of its rarity and the diagnostic challenges associated with this condition [3].

The pathogenesis is incompletely understood and likely involves interactions between genetic susceptibility, abnormal drug metabolism, and viral reactivation, particularly HHV-6 [2,4].

In most patients, it typically begins as a maculopapular eruption that may progress to confluent erythema, with possible features such as purpura, infiltrated plaques, pustules, exfoliative dermatitis, or target-like lesions. Systemic manifestations include fever (≥38.5°C), lymphadenopathy, and hematologic abnormalities (e.g., leukocytosis, eosinophilia, atypical lymphocytosis), along with signs of visceral involvement. At least one internal organ is affected in ~90% of cases; ~35% have involvement of two organs, and up to 20% have multi-organ involvement [4]. Internal organ involvement may precede the onset of rash, and its course may not parallel cutaneous findings. Liver involvement is the most common manifestation (53-90%), usually mild and transient, with cholestatic (37%), hepatocellular (19%), or mixed (27%) patterns. Kidney involvement is variable and includes proteinuria, hematuria, interstitial nephritis, and acute kidney failure. Acute interstitial nephritis occurs in 10-30% of cases, while up to 8% develop renal failure and ~3% may require dialysis. Other organs (less commonly) may be affected, including the lungs, heart, nervous system, and gastrointestinal tract, and some patients may present with SIRS (systemic inflammatory response syndrome)-like features [1,4]. Despite the name of this syndrome, skin involvement may be absent, and eosinophilia is not a mandatory diagnostic criterion; therefore, the condition is also termed drug-induced hypersensitivity syndrome (DIHS) [2]. Diagnosis is commonly supported by the RegiSCAR scoring system, which standardizes the classification of DRESS cases based on clinical, laboratory, and organ involvement criteria [4]. It is important to differentiate DRESS from other diseases that involve the skin, such as viral exanthems, which may also present with systemic symptoms and can be accompanied by peripheral eosinophilia but typically lack the characteristic drug-exposure history and multi-organ involvement seen in DRESS. Similarly, systemic lupus erythematosus, Kawasaki disease, and scalded skin syndrome should be considered in the differential diagnosis based on clinical context and laboratory findings. It is particularly important to distinguish DRESS from other severe drug-related skin reactions such as SJS (Stevens-Johnson syndrome) and TEN (toxic epidermal necrolysis), which are clinically characterized by a shorter latency period and the presence of necrotic keratinocytes and necrosis of the epidermis [2].

We report a case of DRESS syndrome following amoxicillin-clavulanate exposure presenting with predominant acute kidney involvement and cholestatic liver injury in the absence of typical hematologic abnormalities, including significant eosinophilia or atypical lymphocytosis. This case highlights the complexity and variability of DRESS syndrome, where tools such as the RegiSCAR scoring system are essential for supporting the diagnosis in atypical clinical presentations.

## Case presentation

A 75-year-old female presented to the emergency department with generalized weakness, fever (39.5°C), chills, and worsening pruritic rash. Approximately three weeks prior to admission, the patient developed respiratory symptoms and started a one-week course of amoxicillin-clavulanate. Her respiratory symptoms resolved within a few days of completing the antibiotic course. However, 10 days later, she noticed the onset of a maculopapular erythematous rash on her trunk, which progressively spread to her back and extremities over the following week, accompanied by fever, pruritus, and malaise. During this period, her symptoms worsened, with pruritus becoming severe and distressing, ultimately prompting hospital admission. The patient denied any additional medication use or known drug allergies.

**Figure 1:**
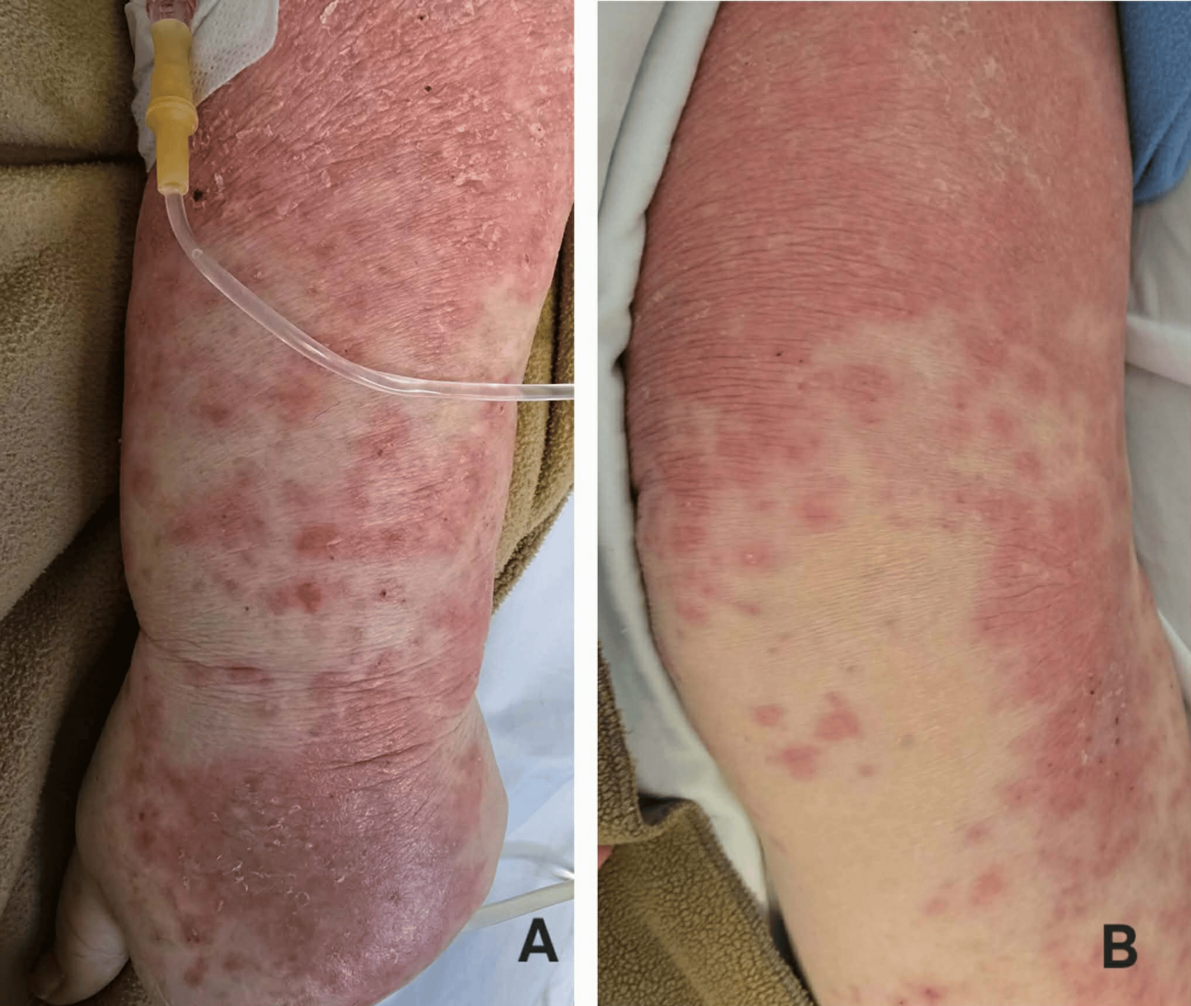
Skin findings on admission (A) Upper extremity showing numerous erythematous macules and papules, partially coalescing, with associated excoriations and mild desquamation. (B) Lower extremity demonstrating more extensive areas of confluent erythema, also with subtle desquamation.

On examination, she was hemodynamically stable: blood pressure 135/86 mmHg, heart rate 98 bpm, and oxygen saturation 98%. She had mild scleral icterus and a generalized erythematous maculopapular eruption involving the trunk, back, and extremities, with areas of confluence and excoriations secondary to intense pruritus (Figure 1), sparing the palms, scalp, and mucous membranes. Mild facial edema was noted. Additionally, bilateral cervical and axillary lymphadenopathy was present, with lymph nodes described as mildly enlarged, mobile, and non-tender.

Laboratory evaluation revealed leukocytosis with neutrophilia and mild eosinophilia (Table 1). Inflammatory markers were elevated, with C-reactive protein (CRP) at 115 mg/L. Renal function showed acute kidney injury with creatinine 278.4 µmol/L and urea 11.77 mmol/L (Table 2). Liver function tests demonstrated a cholestatic pattern, with alanine aminotransferase (ALT) 53 U/L, aspartate aminotransferase (AST) 31 U/L, gamma-glutamyl transferase (GGT) 386 U/L, and total bilirubin 28 µmol/L (Table 2). The urinalysis revealed proteinuria and hematuria (Table 3).

**Table 1:** Complete blood count and inflammatory markers on admission WBCs: White blood cells; CRP: C-reactive protein

Parameter	Result	Reference Range	Interpretation
WBCs (×10⁹/L)	11.65	4.00–11.00	↑ Elevated
Neutrophils (×10⁹/L)	9.36	2.00–7.00	↑ Elevated
Atypical lymphocytes (×10⁹/L)	0.01	≤ 0.20	Normal
Eosinophils (×10⁹/L)	0.34	0.10–0.25	↑ Elevated
CRP (mg/L)	115	<5	↑ Elevated

**Table 2:** Renal and liver function on admission GGT: Gamma-glutamyl transferase; ALT: Alanine aminotransferase; AST: Aspartate aminotransferase

Parameter	Result	Reference Range	Interpretation
Creatinine (µmol/L)	278.4	45–84	↑ Elevated
Urea (mmol/L)	11.77	2.76–8.07	↑ Elevated
GGT (U/L)	386	5–36	↑ Elevated
ALT (U/L)	53	≤35	↑ Slightly elevated
AST (U/L)	31	≤32	Normal
Total Bilirubin (µmol/L)	28	≤24	↑ Slightly elevated

**Table 3:** Urinalysis on admission

Parameter	Result	Reference Range	Interpretation
Protein	100 mg/dL	Negative	↑ Mild proteinuria
Blood	Moderate	Negative	↑ Hematuria
Leukocytes	Negative	Negative	Normal
Nitrites	Negative	Negative	Normal

In the context of liver involvement and systemic features, a targeted workup including autoimmune serology (antinuclear antibodies (ANA), anti-smooth muscle antibodies (ASMA), antimitochondrial antibodies (AMA), and anti-double-stranded DNA antibodies (anti-dsDNA)) and viral hepatitis screening (hepatitis B surface antigen (HBsAg) and anti-hepatitis C virus antibodies (anti-HCV)) was performed, with all results negative. Blood cultures were likewise negative, showing no bacterial growth. A skin biopsy was considered; however, it was not performed due to lack of patient consent. Based on the laboratory findings, a drug-induced etiology remained the most likely cause of the observed clinical syndrome, with DRESS syndrome suspected given the combination of cutaneous, renal, and hepatic manifestations.

Given the widespread cutaneous involvement and severe pruritus causing significant sleep disturbance, systemic corticosteroid therapy was initiated with intravenous dexamethasone (4 mg three times daily). This was complemented by adjunctive high-potency topical corticosteroids applied to affected areas and oral antihistamines to alleviate pruritus.

**Figure 2:**
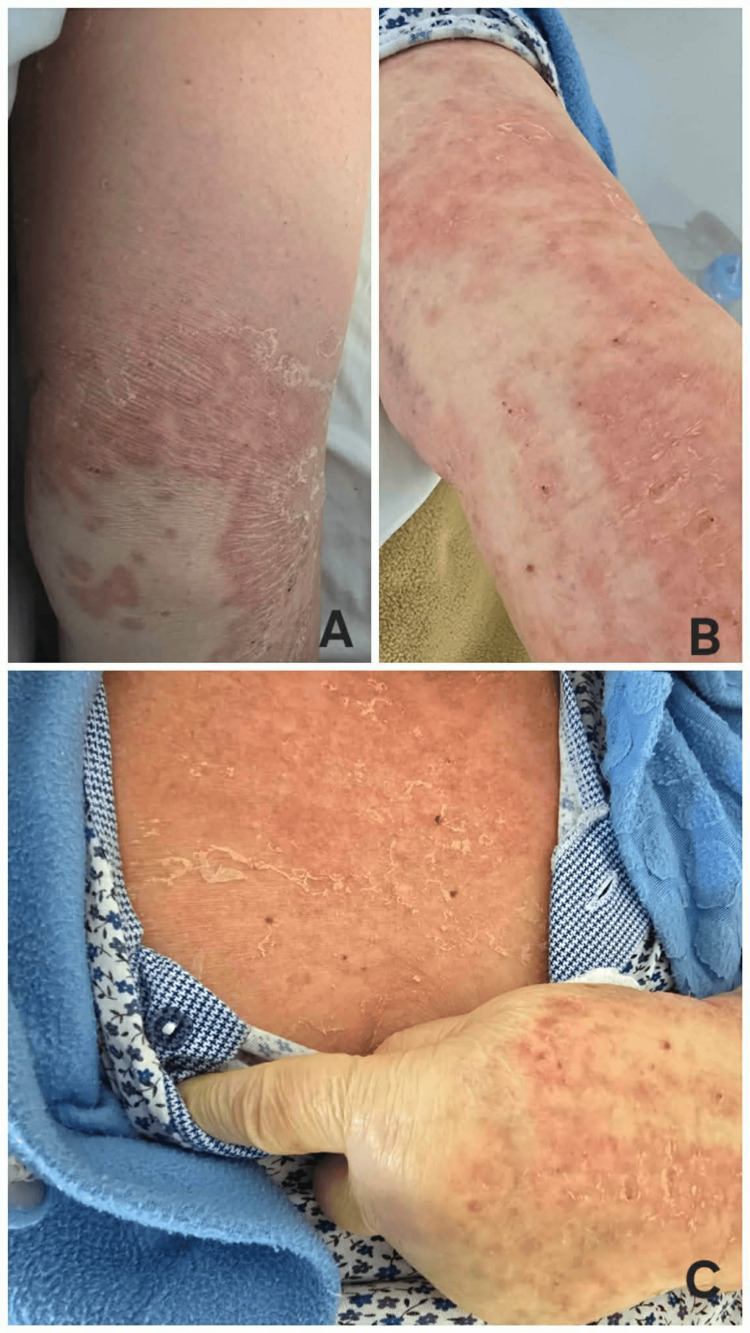
Skin findings on hospital day 9 Residual erythematous rash with prominent desquamation, representing the resolving phase of DRESS syndrome, as demonstrated on the lower extremity (A), upper extremity (B), and chest (C).

The patient’s fever exhibited a rapid decline and had fully resolved by hospital day 7. During this period, her predominant symptom remained intense pruritus, which continued to significantly disrupt sleep despite administration of both systemic and topical therapies. By hospital day 9, there was a notable attenuation of pruritus, accompanied by substantial regression of the cutaneous eruption, with residual lesions primarily consisting of prominent desquamation (Figure 2).

**Figure 3:**
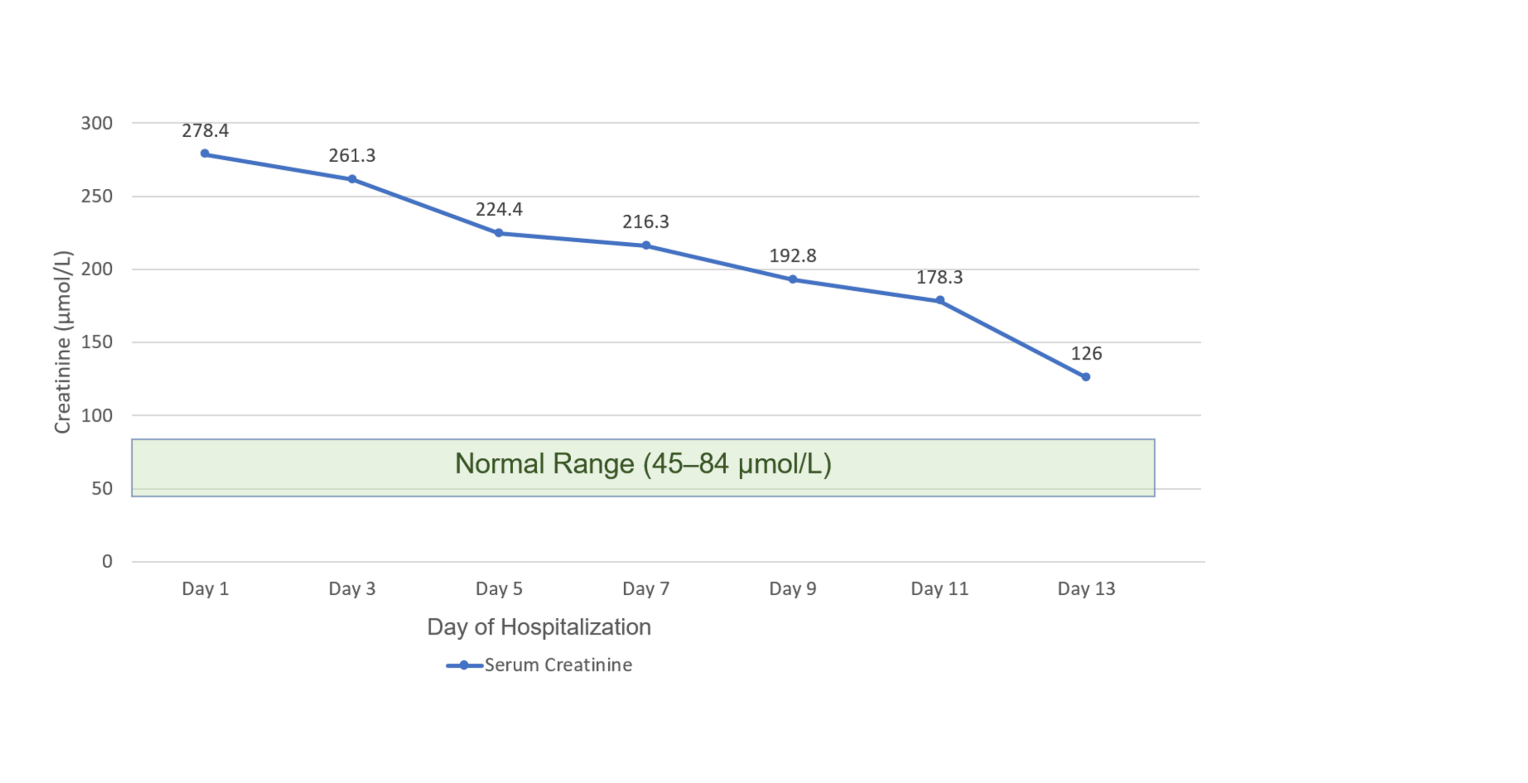
Dynamic changes in serum creatinine during hospitalization

**Figure 4:**
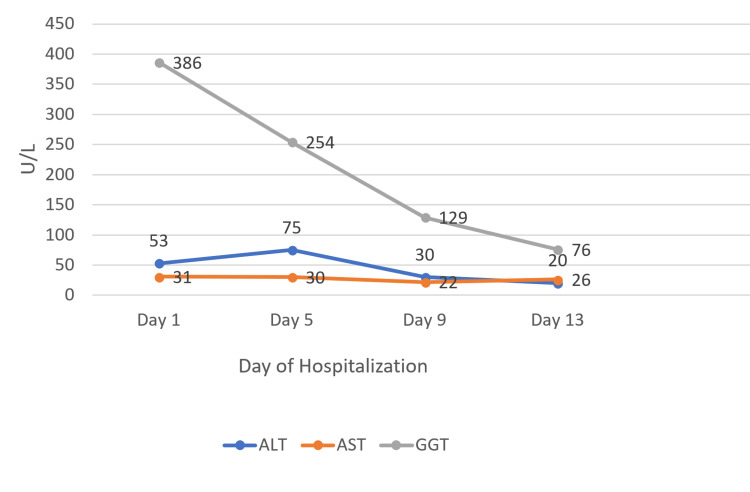
Dynamic changes in serum liver enzymes during hospitalization ALT and AST levels exhibited mild fluctuations throughout the observation period. GGT showed a rapid decline from its initial elevation, indicating transient cholestatic activity.

On day 13, she was afebrile, hemodynamically stable, and free of active skin lesions, with only residual desquamation remaining. The RegiSCAR score of 7 supported a definite diagnosis of DRESS syndrome, which was further reinforced by the clear clinical and laboratory improvement observed under systemic corticosteroid therapy. Renal function had substantially improved, with serum creatinine decreasing to 126 µmol/L (Figure 3), although it remained above the normal range. The patient demonstrated clear clinical improvement, accompanied by a decline in inflammatory markers (CRP 21 mg/L). Laboratory findings showed mild leukocytosis with neutrophil predominance (Table 4), likely reflecting the systemic effects of corticosteroid therapy rather than ongoing infection. Urinalysis was unremarkable. Liver function tests demonstrated a marked decline in GGT, although levels remained slightly elevated (Figure 4). She was discharged on a tapering course of oral corticosteroids with outpatient follow-up.

**Table 4:** Laboratory findings on day 13 of hospitalization

Parameter	Result	Reference Range	Interpretation
WBC (×10⁹/L)	12.27	4.00–11.00	↑ Elevated
Neutrophils (×10⁹/L)	17.18	2.00–7.00	↑ Elevated
Atypical lymphocytes (×10⁹/L)	0.03	≤ 0.20	Normal
Eosinophils (×10⁹/L)	0.02	0.10–0.25	↑ Elevated
CRP (mg/L)	21	<5	↑ Elevated

## Discussion

The diagnosis of DRESS in the present case was established using the RegiSCAR scoring system, which remains one of the most widely accepted tools for standardizing diagnosis in both clinical practice and research settings [4,5]. Our patient achieved a cumulative score of 7 (Table 5) [4], meeting criteria for a definite diagnosis of DRESS. Key contributing features included fever, lymphadenopathy, extensive cutaneous involvement with facial edema, and renal and cholestatic liver involvement. Despite the absence of specific allergy testing or objective confirmation of drug hypersensitivity, the association was classified according to the WHO-UMC system as "probable/likely" based on a reasonable temporal relationship, known association of the drug with DRESS syndrome, and exclusion of alternative causes.

**Table 5:** RegiSCAR score applied to the case Total score: <2: Excluded; 2-3: Possible; 4-5: Probable; ≥6: Definite [4]

Clinical Parameters	Score (-1)	Score (0)	Score (1)	Comments	Points Assigned
Fever ≥101.3°F (38.5°C)	No/unknown	Yes	-	-	0
Lymphadenopathy	-	No/unknown	Yes	>1 cm, at least 2 sites	1
Eosinophilia ≥0.7 × 10⁹ or ≥10% if leukopenia	-	No/unknown	Yes	Score 2 points if ≥1.5 × 10⁹	0
Atypical lymphocytes	-	No/unknown	Yes	-	0
Skin rash					
Rash suggestive of DRESS	No	Unknown	Yes	≥2: facial edema, purpura, infiltration, desquamation	1
Extent ≥50% of BSA	-	No/unknown	Yes	-	1
Skin biopsy suggestive of DRESS	No	Yes/unknown	-	-	0
Organ involvement	-	No	Yes	1 point per organ, max 2	2
Disease duration ≥15 days	No/unknown	Yes	-	-	1
Exclusion of other causes	-	No/unknown	Yes	1 point if ≥3 tests negative (HAV, HBV, HCV, mycoplasma, chlamydia, ANA, blood culture)	1

A retrospective analysis of 72 DRESS cases demonstrated hepatic involvement in the majority of patients (86.1%), with cholestatic injury being the most frequent pattern (35.5%), followed by mixed (29.0%) and hepatocellular (19.4%) types [6,7]. In our patient, liver function tests demonstrated a cholestatic pattern with markedly elevated GGT and mild hyperbilirubinemia, consistent with these previously reported frequencies.

A systematic review of 89 articles, including 56 case reports and 33 cohort studies, evaluated HHV-6 reactivation in 748 DRESS patients. Reactivation was detected in 470 patients (62.8%, 95% CI 0.59-0.66), significantly higher than other herpesviruses such as CMV (24.7%), EBV (30.4%), and HHV-7 (16.0%). HHV-6 reactivation typically occurs two to four weeks after symptom onset and has been associated with longer hospital stays, increased fever duration, more disease flares, and higher rates of long-term sequelae compared with HHV-6-negative patients [8].

Japanese authors have proposed including HHV-6 reactivation as a diagnostic criterion [5], although this is not yet universally accepted. The pathogenesis may involve drug-triggered viral replication, leading to immune activation via viral antigens and extensive antiviral T cell responses. EBV and other herpesviruses may play a similar role, but HHV-6 appears particularly relevant, as drugs like amoxicillin and sodium valproate can directly stimulate HHV-6 replication [9]. In our patient, amoxicillin-clavulanate could have similarly triggered viral reactivation, potentially contributing to the renal and hepatic manifestations; however, this remains speculative, as viral testing was not performed and reactivation was not confirmed.

Without such testing, it is not possible to determine whether viral reactivation contributed to the severity of organ involvement in this patient. Additional limitations include incomplete eosinophil counts and liver enzyme measurements (ALT, AST), along with the absence of atypical lymphocytosis, as well as the lack of drug rechallenge testing, leaving some uncertainty regarding the exact causative agent. These constraints reflect the practical challenges in acute clinical settings. Despite these limitations, the overall clinical course, temporal relationship with drug exposure, and response to therapy strongly support the diagnosis. Discontinuation of the offending drug is essential in DRESS management, and systemic corticosteroids remain the mainstay of treatment, while topical steroids may be sufficient in mild cases [10].

Renal involvement in DRESS syndrome must be distinguished from other causes of eosinophilia and acute kidney injury. Acute interstitial nephritis (AIN) is characterized by inflammation in the kidney interstitium and can arise from drugs, autoimmune diseases, infections, or idiopathic causes. Drug-induced AIN (DI-AIN) accounts for up to 85% of AIN cases, most commonly due to NSAIDs, proton-pump inhibitors, and antibiotics [11]. Clinically, DI-AIN may resemble DRESS, presenting with rash, fever, and eosinophiluria. However, DRESS typically involves additional organs, such as the liver or lungs, and is often accompanied by features like lymphadenopathy and facial edema, which are absent in DI-AIN. In these situations, calculating the RegiSCAR score and reviewing the drug exposure timeline are essential, as DRESS generally has a longer latency period than other severe cutaneous adverse reactions (SCARs). Renal manifestations in patients with DRESS syndrome include elevated creatinine, reduced glomerular filtration rate, oliguria, anuria, proteinuria, and hematuria. Notably, not all patients develop acute kidney injury or show creatinine elevation; some may present with isolated proteinuria or hematuria [12].

In our patient, renal involvement was evident as acute kidney injury with elevated creatinine and urea, accompanied by proteinuria and hematuria, which improved progressively after corticosteroid therapy, illustrating the typical spectrum and reversibility of renal manifestations in DRESS. Although drug-induced interstitial nephritis (DI-AIN) can present with acute kidney injury and typically responds to corticosteroids, the temporal gap between cessation of oral amoxicillin-clavulanate and the onset of renal impairment in this patient argues against direct nephrotoxicity. Nephrotoxicity associated with this antibiotic typically presents as acute interstitial nephritis or crystal nephropathy and is more frequently linked to intravenous rather than oral administration [13]. Moreover, characteristic urinary markers of DI-AIN, such as leukocyturia, eosinophiluria, or granular casts, were absent. The concurrent presence of systemic manifestations, including rash, fever, lymphadenopathy, and cholestatic liver injury, supports that renal involvement was primarily mediated by the systemic hypersensitivity reaction characteristic of DRESS syndrome rather than by direct nephrotoxic effects of the antibiotic.

## Conclusions

This case illustrates an uncommon presentation of DRESS syndrome with predominant renal involvement and cholestatic liver injury following amoxicillin-clavulanate exposure. Despite the absence of key hematologic features, including atypical lymphocytosis and eosinophilia meeting diagnostic thresholds, the diagnosis was established using the RegiSCAR scoring system, underscoring the importance of structured criteria in recognizing incomplete or diagnostically challenging presentations. In our patient, the temporal relationship to drug exposure served as an important, albeit non-confirmatory, diagnostic clue; however, such timelines are often unclear or unavailable in clinical practice, which may further complicate assessment. Given the broad differential diagnosis, variable clinical presentation, and lack of a single definitive diagnostic test, a high index of suspicion and careful clinical evaluation remain essential for accurate diagnosis and timely management. Although not performed in our case, additional consideration should be given to potential viral reactivation, particularly human herpesvirus 6 (HHV-6), which is a proposed but not consistently demonstrable factor in DRESS pathogenesis. This is particularly important in cases with predominant renal involvement, which may mimic alternative nephrological conditions, where delayed recognition may adversely affect patient outcomes.
